# Characterization of Fatty Acid Photodecarboxylase in Zeolitic Imidazolate Frameworks

**DOI:** 10.1021/acsomega.5c01397

**Published:** 2025-08-06

**Authors:** Min-Shih Su, Ya-Ting Kao

**Affiliations:** † Department of Biological Science and Technology, College of Engineering Bioscience, 34882 National Yang Ming Chiao Tung University, Hsinchu 30068, Taiwan; ‡ Institute of Bioinformatics and Systems Biology, College of Engineering Bioscience, National Yang Ming Chiao Tung University, Hsinchu 30068, Taiwan; § Center For Intelligent Drug Systems and Smart Bio-devices (IDS^2^B), National Yang Ming Chiao Tung University, Hsinchu 30068, Taiwan

## Abstract

Fatty acid photodecarboxylase (FAP) was recently discovered in microalgae and is a photoenzyme that harvests sunlight to convert long-chain fatty acids to hydrocarbons. The hydrophobicity of the substrates and photoproducts strongly affects the stability of FAP *in vitro* studies. Here, we incorporated zeolitic imidazole frameworks (ZIFs) as structural protecting cages and characterized *Chlorella variabilis* FAP (*cv*FAP) in ZIFs. Due to the preparation conditions and the surface properties, ZIFs with a hydrophobic surface (ZIF-8) form a cluster-like packed morphology, and ZIFs with a hydrophilic surface (ZIF-90) form a hexagon-like morphology. However, both ZIFs compress FAP and lead to a more hydrophobic active-site environment and fewer active-site water molecules. The ZIF-90 cages enhance the *cv*FAP activity at higher temperature conditions. Upon irradiation, FAP undergoes energy deactivation and photoinactivation, and these processes compete with each other. In both ZIF cages, the deactivation processes of the excited FAP are slightly enhanced, resulting from the bent cofactor conformation. Although the ZIFs provide protective confinement to stabilize the enzyme structures when structural destruction originates from molecular fragmentation casing by photoinduced radical reactions, such protective effects are limited.

## Graphical abstract

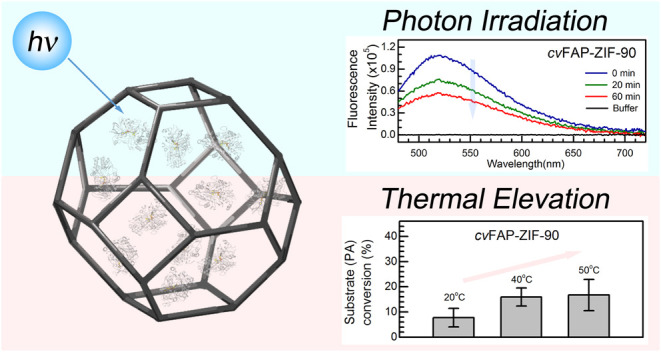

## 1 Introduction

Photoenzymes are a rare type of catalyst, and their catalytic functions are light-driven.^1–7^ Fatty acid photodecarboxylase (FAP) is one of the three photoenzymes and was recently discovered in microalgae.^1,2^ In the cell cultures of *Chlamydomonas reinhardtii* (Chlorophyceae), *Chlorella variabilis* (Trebouxiophyceae), and several *Nannochloropsis* species (Eustigmatophyceae), long-chain hydrocarbons were detected resulting from the decarboxylation of the corresponding fatty acids.^1^ FAP contains a flavin adenine dinucleotide (FAD) cofactor and harnesses the blue light to drive the decarboxylation of the long-chain fatty acid substrates (C_12_–C_18_) to form products of C_*n*–1_ alkane/alkene. Under blue-light irradiation, a proposed mechanism involves an excited-state electron transfer (ET) from the substrate to the excited-state flavin cofactor.^2,8–10^ Upon electron transfer, a radical-pair intermediate (anionic semiquinone FAD^•–^–fatty acid radical RCOO^•^) forms, and the cofactor should restore its original redox state to complete the catalytic cycle and restart a new turnover. The decarboxylated substrate should incorporate an additional hydrogen atom or proton to form its corresponding hydrocarbon product. Hence, the subsequent steps should involve an electron return from the product to the cofactor, possibly coupling with a hydrogen atom or proton transfer.^2,8–10^

Although the photoefficiency of decarboxylation is high, the inactivation processes were observed in several *in vitro* studies.^11,12^ Upon photoexcitation, the photoexcited and high redox-potential ^1^FAD* could be an electron acceptor and oxidize nearby amino acid residues, causing irreversible inactivation of the *cv*FAP.^11^ The excited ^1^FAD* could undergo intersystem crossing, and the excited triplet ^3^FAD* is generated, resulting in reactive oxygen species (ROS) production.^13^ Either by forming oxidized amino acids or reactive oxygen species, radicals are generated and enzymes are destabilized. Hence, several approaches have been performed to attenuate the photoinactivation effects by adding fatty acids^12^ or keeping anaerobic conditions.^13^ In the presence of fatty acids, the photocatalytic processes compete with the photoinactivation, and an electron is transferred from the substrate to initiate the catalytic reaction and suppress the photoinactivation processes. Hence, modulating the excited ^1^FAD* decay pathways would possibly stabilize the enzyme activity or decrease the inactivation occurrence.

The hydrophobicity of substrates and photoproducts disturbs the stability of *cv*FAP. Under blue-light irradiation, the accumulation of photoproducts occurs. The low solubility of the long-chain alkane/alkene photoproducts strongly affects the stability of *cv*FAP. In several *de novo* approaches,^14–17^ metal–organic frameworks (MOFs) provide protective layers for proteins by reducing the structural changes and further protein unfolding. The MOFs that were constructed from Zn^2+^ and imidazole (Im) ligands exhibit zeolitic-like properties and are known as zeolitic imidazole frameworks (ZIFs). The fast synthesis of hierarchical porous ZIFs has been demonstrated at room pressure and temperature in environmentally friendly approaches.^18–23^ In previous studies on catalases (CAT), from bovine liver, the CAT@ZIF-90 composites from mixing the ZIF precursors with catalases exhibit behavior different from that of CAT-on-ZIF-90, and these results demonstrate that the catalase molecules are embedded in the ZIF-90 supports, not on the surface of ZIF-90. The CAT@ZIF-90 composites act on the substrate hydrogen peroxide to form the products water and oxygen even in harsh conditions.^16^ Several studies showed the modification either on surface properties or framework structures of ZIFs or even on the enzyme structures to optimize bioactivity.^23,24^ Here, we incorporated ZIFs as the protective coating to stabilize the *cv*FAP in an aqueous solution. We selected two ZIFs, ZIF-8 and ZIF-90, with distinct surface properties and systematically characterized photocatalysis, photoinactivation, and excited-state dynamics of *cv*FAP in aqueous solution and biocomposites.

## 2 Materials and Methods

### 2.1 Plasmid Design and Protein Expression and Purification

The *C. variabilis* (NC64A) FAP sequence (*cv*FAP Uniprot: A0A248QE08) comprising the FAD-binding domain and the substrate-binding domain (62–654 bp), but without the residue transit peptide (TPs, 1–61 bp),^2^ was designed from GenBank (KY511411.1) with further codon optimization. The codon-optimized *cv*FAP was subcloned into the *Nde*I and *Hin*dIII sites of the pET28a vector. The pET28a-*cv*FAP construct was used to express *cv*FAP as in the previous report, with some modifications.^2,8,9^ Briefly, *Escherichia coli* BL21 (DE3) pLysS cells containing the plasmid pET28a-*cv*FAP were grown at 37 °C in LB broth containing 50 mg/L kanamycin to the absorption of 0.6 at 600 nm and induced at 17 °C for 18–24 h with 1 mM isopropyl-β-d-1-thiogalactopyranoside and 400 nM flavin FAD standard (Sigma F8384). After harvesting, the *cv*FAP enzymes were purified by the liquid chromatography system with HisTrap HP columns (Ni Sepharose affinity resin). With a gradient elution from 10 to 500 mM imidazole in 50 mM Tris buffer containing 500 mM NaCl and 20% glycerol at pH 8.0, *cv*FAP was eluted between 150 and 255 mM imidazole. We carefully kept the expression and purification processes with minimal light exposure to maximize the expression level of the active and stable enzymes.^25,26^ The *cv*FAP was dialyzed in a storage buffer of 50 mM Tris, 500 mM NaCl, and 50% glycerol at pH 8.0. To minimize the photoinactivation effect, during the sample preparation, including cell culture, sonication, purification, and dialysis, samples were kept in the dark.

### 2.2 Preparation of the Biocomposite System with Zeolitic Imidazole Frameworks

For the synthesis of the biocomposite, ZIF precursors were mixed with *cv*FAP. These precursors formed ZIF and *cv*FAP enzymes were encapsulated inside the ZIF cages instead of squeezing through the pores into the ZIF cages. For the synthesis of the *cv*FAP-ZIF-8 biocomposite, the 2-methylimidazole (2-MIM) aqueous solution (solution A_1_: 1.4 M and 4.2 mL) and the protein solution (*cv*FAP: 29.2 μM and 3 mL) were mixed and kept at 4 °C for 5 min. The Zn­(NO_3_) aqueous solution (solution B_1_: 40 mM and 4.2 mL) was added to the previous mixture by slowly stirring (130 rpm) at 4 °C for more than 1 h. The synthesized *cv*FAP-ZIF-8 biocomposite forms submicrometer particles and precipitates. The precipitates of the *cv*FAP-ZIF-8 biocomposite were collected by a microcentrifuge (5000 rpm, 3 min) and washed with water for at least five cycles to remove enzyme aggregation and free FAD molecules.^16,20^ For the synthesis of the *cv*FAP-ZIF-90 biocomposite, 50 mg of poly­(vinylpyrrolidone) (PVP) was added to the imidazole-2-carboxaldehyde (ICA) aqueous solution (solution A_2_: 200 mM and 25 mL) and heated to 70 °C in a water bath with continuous stirring (130 rpm) until the chemicals dissolved. The mixed solution was gradually cooled to 45 °C, and the protein solution (*cv*FAP: 29.2 μM and 3 mL) was added. The mixture solution was further mixed with the Zn­(NO_3_) aqueous solution (solution B_2_: 400 mM and 3 mL) at 43 °C for 5 min. The precipitates of the *cv*FAP-ZIF-90 biocomposite were collected immediately by a microcentrifuge (5000 rpm, 3 min) and washed with water for at least five cycles.^18,21^ A high temperature of 70 °C is required for well-dissolving PVP in an ICA/H_2_O solution. The ICA molecules could be distributed evenly in the solution, and higher-concentration metal ions could quickly target ICA molecules. In addition, the higher temperature of 43 °C is used to avoid the quick aggregation of ZIF-90 cages. Hence, the temperature requirement, concentration of metal ions, and harvesting time are crucial in the uniform formation of ZIF-90 cages.^22^ Moreover, the ratio of precursors (2-MIM or ICA) and metal ions affects the size of the ZIF cages.^22,23^ A schematic illustration of biocomposite synthesis is shown in Figure S1. Both biocomposites were stored at 4 °C and resolved with the storage buffer (50 mM Tris, 500 mM NaCl, and 50% glycerol) before subsequent experiments.

### 2.3 Characterization of *cv*FAP-ZIF-8 and *cv*FAP-ZIF-90 Biocomposites

The morphologies of *cv*FAP-ZIF-8 and *cv*FAP-ZIF-90 biocomposites were examined by optical microscopy with both differential interference (DIC) and confocal fluorescence images (Leica TCS-SP5-X AOBS). Both samples were loaded in a Petri dish and excited by a white light laser in the 470–480 nm range. The fluorescence images were measured by collecting 500–800 nm fluorescence through a 63× magnification objective. All images were enhanced with three superimposed scans and were processed by Leica LAS-AF-lite software. Furthermore, we used a super-resolution microscope equipped with lattice-structured illumination (Zeiss Elyra 7 with Lattice SIM^2^) to confirm the enzyme encapsulation by the ZIF cages. The *cv*FAP-ZIF-90 biocomposites were excited at 488 nm, and fluorescence images were taken in the stacked mode with an sCMOS camera. The acquired images were processed with classic SIM for enhanced resolution by the ZEN 3.0 microscopy software.

### 2.4 Enzyme Activity Assays

The activity of *cv*FAP was determined at 25 °C by following the decrease of substrates and increase of products using the fluorometric method (BioVision K612, the ACS-ACOD assay) and gas chromatography–mass spectrometry (GC-MS). The concentrations of substrates were also determined by the fluorometric method compared with a standard palmitic acid (PA) solution, which was provided along with the assay. In the assay, substrates were converted to their acyl-coenzyme A (CoA) derivatives and subsequently oxidized to the end product with the concomitant fluorescence generation (Ex535 nm/Em585 nm). The simplified reaction scheme for the assay is described in the Supporting Information and shown in Figure S2. We also performed the blank control examination with only *cv*FAP under blue-light irradiation. Hence, the unreacted substrates were precisely quantified by a fluorometric method. The structures of the substrate and photoproduct were confirmed by mass spectrometry, and their amounts were estimated from the area of the corresponding retention time in gas chromatography.

The substrates at various concentrations (0, 0.5, 1.0, 1.5, 2.0, and 2.5 μM) react with the reagents from the ACS-ACOD kit to generate the standard curve. The *cv*FAP-substrate complexes were first prepared in the dark with 7 μM *cv*FAP and 200 μM palmitic acid (PA) substrate in the reaction buffer containing 40 mM Tris, 400 mM NaCl, 40% glycerol, and 20% dimethyl sulfoxide (DMSO) at pH 8.0. The 20% DMSO in the reaction buffer is essential to increase the solubility of the substrate and its corresponding alkane photoproduct. The concentrations of compounds in solutions were determined by UV–vis absorbance using the molar extinction coefficients: oxidized FAD (ε_467_ = 11 300 M^–1^ cm^–1^; ε_280_ = 24 300 M^–1^ cm^–1^) and *cv*FAP (ε_280_ = 65 695 M^–1^ cm^–1^).^11^ The 450 nm laser beam of 10 mW was reshaped by a cylindrical lens and focused into a quartz cuvette containing the complex sample. Every selected time, a tiny portion (10 μL) of the complex sample was taken out and kept in the dark to stop the photoreaction by heat shock processes for 10 min at 90 °C. Due to the high concentration of PA in the enzyme activity assay, a 10 μL *cv*FAP-PA complex sample was taken out at various irradiation times, and a 40-fold dilution was used to quantify PA concentration in the linear range of the standard curve.

Upon 535 nm excitation, the 585 nm fluorescence spectrum of each sample was recorded. The 585 nm emission intensity exhibits a linear relation with the concentration of substrate. Hence, the concentration of unreacted substrates in the 10-μL complex samples was determined by the 585 nm emission intensity after the ACS-ACOD assay reaction. Hence, we determined the time trace of the substrate consumption under increasing light irradiation. The stationary absorption and fluorescence spectra were recorded with a spectrophotometer (U-3900, Hitachi High-Tech.) and a fluorimeter (FluoroMax4, HORIBA Jobin Yvon Inc.).

The complex samples with the 450 nm irradiation time of 0 and 4 h underwent heat shock processes for 10 min at 90 °C to stop the catalytic reaction. Multiple ether extractions were performed to remove DMSO, which causes interference in the GC-MS analysis. Samples were further transferred to a centrifuge at 13 000 rpm for 5 min, and the supernatants were collected. Finally, the solvents were further low-pressure dried. Then, samples were redissolved in 100 μL of ether and were heated by a pyrolyzer (FRONTIER, EGA/PY-3030D) into the GC-MS (Agilent 7890CB/JEOL Ltd. AccuTOF GCx) with the separation column (Rxi-5MS) and Helium as carrier gas at a flow rate of 1 mL/min at 300 °C after electron ionization.

### 2.5 Nanosecond Fluorescence Decay

The nanosecond fluorescence decay was measured by a time-correlated single-photon counting (TCSPC) system. Protein samples were excited by an 80 MHz fs laser pulse (MaiTai Ti:Sapphire ultrafast lasers, Spectra-Physics) equipped with a doubling system (Inspire-Blue automated harmonic generators). The excitation wavelength of 450 nm was used, and the peak-emission wavelengths were chosen for detection by a double-additive monochromator (9030DA, Sciencetech). The polarization between the blue-light excitation and fluorescence emission was oriented at a magic angle (54.7°). The TCSPC module (PicoHarp300, PicoQuant) was used to obtain the histogram of the fluorescence signal over time. The time resolution was limited by the photodetector (PD-100-CTC, MPD) with an instrument response time of 35–50 ps. For the fluorescence lifetime of the FAD chromophore, protein samples were excited at 450 nm and the 560 nm fluorescence decays were measured. The fluorescence decays were analyzed globally with multiexponential decays by the software (EasyTau2, PicoQuant).

## 3 Results and Discussion

### 3.1 Characterization of *cv*FAP and *cv*FAP-ZIF Biocomposites

The absorption, excitation, and emission spectra of *cv*FAP in the reaction buffer and two ZIFs, along with oxidized FAD in the reaction buffer, are shown in Figure 1. We measured the fluorescence quantum yield of FAD using a comparative method with rhodamine 6G as the reference standard. The fluorescence quantum yields of FAD were 0.04 and 0.13 in the reaction buffer and the *cv*FAP protein environment, respectively, and these results are similar to those of the oxidized flavin in other systems.^27–30^ Due to the open structure of FAD in the *cv*FAP protein environment, FAD exhibits higher fluorescence quantum yields.^27^ Based on the absorption at 280 and 467 nm, we estimated that approximately 75% of purified *cv*FAP contains the FAD cofactor (Figure S3A). Upon excitation by 450 nm, the *cv*FAP in the reaction buffers shows an emission peak of 545 nm. Since the ZIF-8 and ZIF-90 form scattering particles, the scattering curve overwhelms the FAD absorption bands in the biocomposites. Hence, only the fluorescence spectra of the *cv*FAP-ZIF-8 and *cv*FAP-ZIF-90 biocomposites are shown in Figure 1B. ZIF-8 cages are not fluorescent, but ZIF-90 cages exhibit a weak 520 nm peak emission, which partially overlaps with FAD emission. A comparison of the spectra of *cv*FAP in the reaction buffer and ZIF-90 cages is shown in Figure S3B. While excited by 450 nm, the *cv*FAP-ZIF-8 and the *cv*FAP-ZIF-90 biocomposites show an emission peak of 525 nm, and the fluorescence intensity originates from the excited *cv*FAP in biocomposites, not from the ZIF-8 and ZIF-90 cages. From the excitation spectra of 560 nm detection, we observed the S_0_ → S_1_ excitation band at 450 and 460 nm peaks in the *cv*FAP-ZIF-8 and *cv*FAP-ZIF-90 biocomposites, respectively. While comparing the flavin emission spectrum of the *cv*FAP in aqueous solution, those in biocomposites show blue-shifted emission peaks. These results indicate a quite different active-site environment for the FAD cofactor in the two biocomposites. We suggest that *cv*FAP is more packed in the ZIF cage, influencing the electronic energy level of the FAD and resulting in fewer water molecules in the active site.^31,32^

**Figure 1:**
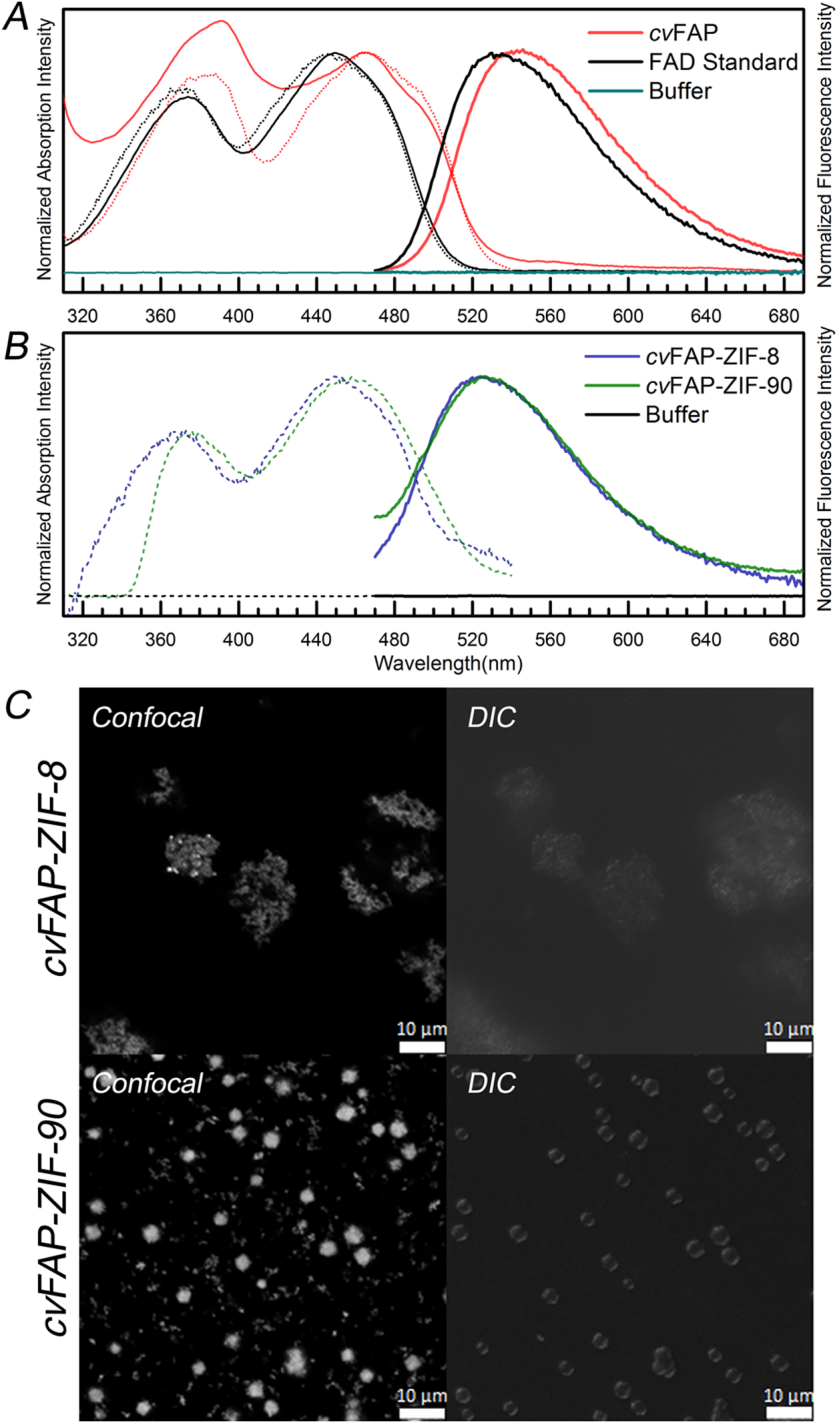
Spectral properties of *cv*FAP in aqueous solution and two biocomposites. (A) The absorption (thin line), emission (bold line), and excitation spectra (short-dotted line) of *cv*FAP in aqueous solution (red), the standard oxidized flavin FAD (black), and the reaction buffer (dark cyan). (B) The emission (bold line) and excitation spectra (short-dotted line) of the *cv*FAP-ZIF-8 biocomposites (blue), the *cv*FAP-ZIF-90 biocomposites (green), and the reaction buffer (black). The ZIF-90 cage emission was removed for clarity. (C) The morphologies of biocomposites by optical microscopy (left: confocal images; right: DIC images). (Upper) The *cv*FAP-ZIF-8 biocomposites and (lower) the *cv*FAP-ZIF-90 biocomposites.

For the synthesis of the biocomposite, ZIF precursors were mixed with *cv*FAP. These precursors form ZIFs, and *cv*FAP enzymes were encapsulated inside the ZIFs matrix. The *cv*FAP is a polypeptide of 654 amino acids and is about 64.9 kDa, similar to bovine serum albumin (BSA, ∼66.5 kDa). In the previous studies on BSA-coumarin-343 bioconjugates and BSA-coumarin-343@ZIF8 biocomposites,^20^ encapsulating the BSA proteins in ZIF-8 indeed reduced the free water molecules due to spatial hindrance but had minor effects on the solvation dynamics of the bound water, indicating that BSA proteins remain in the nature-folded form. The observed sizes of *cv*FAP-ZIF-8 biocomposites are also similar to those of BSA-coumarin 343@ZIF8 biocomposites of 1–2 μm. In the previous studies on catalase (CAT),^14^ catalase is about ∼60 kDa. The CAT@ZIF-90 composites from mixing the ZIF precursors with catalases are embedded in the ZIF-90 supports and not on the surface of ZIF-90.

We examined the morphologies of *cv*FAP-ZIF-8 and *cv*FAP-ZIF-90 biocomposites with DIC and confocal fluorescence microscopy. The DIC images show the morphologies of both cages, and the fluorescence confocal images illustrate the *cv*FAP embedded inside these cages. ZIF-8 cages are not fluorescent, which results in dark fluorescence images. In Figure 1C, the fluorescence image of *cv*FAP-ZIF-8 composites is observed, and this image overlaps with the corresponding DIC image, indicating the colocalization of *cv*FAP enzymes and ZIF-8 cages (Figure S4). If the *cv*FAP enzymes move freely in the buffer and not inside the cages, we would observe an evenly bright fluorescence image. The *cv*FAP-ZIF-8 biocomposites exhibit an average diameter of ∼1–2 μm, and the *cv*FAP-ZIF-90 biocomposites exhibit an average diameter of 3–5 μm. Based on the 2-MIM/zinc ion and the ICA/zinc ion ratio, our observations are similar to those in the previous studies.^20–23^ The increasing diameter and increasing number of larger ZIF-90 cages were observed during image measurements in the *cv*FAP-ZIF-90 biocomposites. This phenomenon could be associated with the surface properties of ZIFs and the preparation conditions. In the *cv*FAP-ZIF-8 biocomposites, ZIF-8 is composed of zinc ions and 2-MIM with a hydrophobic surface and forms aggregations quickly at 4 °C. Hence, we observed a cluster-like packed morphology. In the *cv*FAP-ZIF-90 biocomposites, ZIF-90 is composed of zinc ions and ICA with a hydrophilic surface. The *cv*FAP-ZIF-90 biocomposites are evenly distributed in the buffer and precipitate slowly at 43 °C, leading to a slower aggregation. Hence, we observed a growing hexagon-like morphology. The DIC and confocal fluorescence images of the ZIF-90 cages are shown in Figure S5. We further examined the *cv*FAP-ZIF-90 biocomposites with super-resolution microscopy, and the results show that the *cv*FAP enzymes are encapsulated in the cavity of the ZIF-90. Although the ZIF-90 cages exhibit a weak 520 nm peak emission, Figures S4 and S5 indicate that the *cv*FAP enzymes are encapsulated in the ZIF-90 cages. Moreover, cofactor FAD is noncovalently bound inside the active site of *cv*FAP. When *cv*FAP enzymes denature, the cofactor FAD would be released and spread all over the buffer, leading to an evenly bright fluorescence image. Hence, *cv*FAP enzymes are embedded inside ZIF cages and not in the denatured forms.

### 3.2 Enzyme Activity Assays

With 535 nm excitation, the emission spectra of the assay end products were recorded using the fluorometric method. The standard curves with various concentrations of the PA substrate are shown in Figure 2A inset (black squares). After 450 nm irradiation, the remaining unreacted PA in the diluted *cv*FAP-PA complex forms the end product by the ACS-ACOD assay, and the corresponding fluorometric spectra are shown in Figure 2A. The longer the irradiation time, the less PA that is left, and the weaker the fluorescence intensity. Based on the standard curve, we could estimate the unreacted PA left in the reaction buffer, as shown in Figure 2A inset (blue circle). After consideration of the 40-fold dilution and the enzyme blank control examination, the unreacted PA under various irradiation times is shown in Figure 2B. Under blue-light irradiation, the remaining unreacted PA in the *cv*FAP-ZIF-8 and the *cv*FAP-ZIF-90 composites were also examined by the ACS-ACOD assay (Figure 2C,D). After the 450 nm light of 10 mW for 2 h irradiation, the PA conversion activity of the *cv*FAP-PA complex in aqueous solution was 97.74 ± 5.06% and those in the *cv*FAP-ZIF-8 and *cv*FAP-ZIF-90 biocomposites were −1.16 ± 5.37 and 11.82 ± 1.84%, respectively.

**Figure 2:**
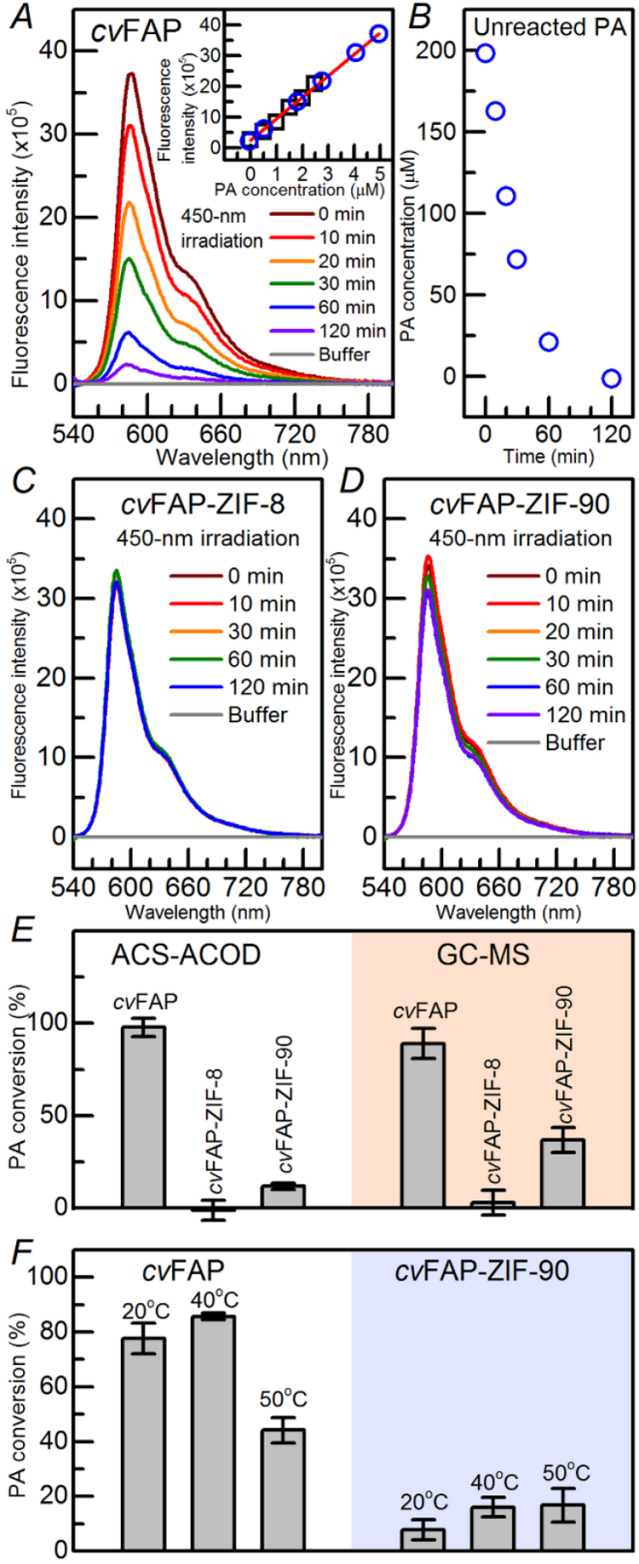
Enzyme activity assay of the *cv*FAP-PA complex in aqueous solution and two biocomposites with PA substrates. (A) The emission spectra of the end product in the ACS-ACOD assay from the *cv*FAP-PA complex upon 450 nm irradiation. The inset shows the standard curves of the PA substrate (black squares) and unreacted PA from the activity assay (blue circles). (B) The unreacted PA substrate under various irradiation times in the aqueous solution. (C, D) The emission spectra of the end product from the *cv*FAP-PA complex in ZIF-8 and ZIF-90, respectively, upon 450 nm irradiation. (E) The bar chart represents the PA-to-PD conversion percentage of *cv*FAP in the aqueous solution and two biocomposites in the ACS-ACOD fluorometric method (left) and GS/MS method (right) at 25 °C. (F) The bar chart represents the PA-to-PD conversion percentage of *cv*FAP in aqueous solution and ZIP-90 biocomposites at various temperatures (20, 40, and 50 °C) by the ACS-ACOD fluorometric method.

The standard substrate PA and product PD retention times are 18.89 and 13.69 min, respectively, in GC. The structures of PA and PD were further confirmed by MS (Figure S7A,B). For product examination in GC/MS, the complex samples with an irradiation time of 0 and 4 h underwent multiple extractions with ether to remove DMSO and were further low-pressure dried to remove solvents. The GC results and the corresponding mass spectra with the analyzed structures are shown in Figure S7C,D. Based on the GC-MS of the *cv*FAP-PA complex under 450 nm irradiation for 0 and 4 h, we observed substrate PA decrease and photoproduct PD increase. With GC-MS, the PA-to-PD conversion performed with *cv*FAP in aqueous solution, ZIF-8, and ZIF-90 was 89.08 ± 8.09, 2.84 ± 6.73, and 36.92 ± 6.72% respectively, as shown in Figure 2E. The PA-to-PD conversions are estimated from the area under the GC peak at about 18.89 min as shown in Figure S8. Due to the uncertainty of multiple extractions and drying procedures, GC-MS was used as a qualitative reference. Hence, we confirmed the structures of PA and PD and the activity of *cv*FAP. The *cv*FAP-ZIF-8 did not perform effective PA conversion activity using either the fluorometric method or GC-MS, but enzyme activity was measured in *cv*FAP-ZIF-90.

Upon binding the substrate, the fluorescence intensity of the cofactor FAD decreases due to the initial catalytic electron transfer, which lowers the fluorescence quantum yield of the excited-state FAD.^2,9,10,33^ In both *cv*FAP in the reaction buffers and the *cv*FAP-ZIF-90 biocomposites, we observed quenching of the excited FAD emission in the presence of the PA substrate (Figure S9), which was not observed in the *cv*FAP-ZIF-8 biocomposites. The loss of fluorescence quenching indicates no additional excited-state decay channel occurring upon PA binding, and more likely, the substrate-binding affinity is extremely low in the *cv*FAP-ZIF-8 biocomposites. Without binding with substrates, the initial electron transfer from substrate to the excited cofactor does not occur, leading to the loss of the PA-to-PD conversion. This is probably due to the hydrophobic surface of ZIF-8, which results in high substrate affinity with the ZIF-8 surface, not with the *cv*FAP inside the ZIF-8 cages. In addition, we observed more stable thermal features in the *cv*FAP-ZIF-90 biocomposites. Even though the conversion percentage is lower in *cv*FAP-ZIF-90 biocomposites, compared to *cv*FAP in the reaction buffer, the ZIF-90 cage protects the *cv*FAP from denaturing and enhances the *cv*FAP activity in higher temperature conditions, as shown in Figure 2F. Thus, the cage protection of ZIF-90 could suppress the environment disturbance in enzyme stability.

### 3.3 Photoinactivation of *cv*FAP and *cv*FAP-ZIF Biocomposites

We observed the loss of the fluorescence intensity of *cv*FAP under high-intensity blue-light irradiation. Such phototoxicity and catalytic instability would limit the investigation of the mechanism and application *in vitro*. As shown in Figure 3A, the fluorescence intensity diminishes by more than 50%, and the emission peak shifts toward 530 nm under 450 nm irradiation for 60 min. The observation indicates protein structure degradation and possible cofactor release. Upon excitation, the excited FAD cofactor involves deactivation and inactivation processes. Through nonradiative processes and radiative fluorescence, the excited FAD undergoes energy relaxation and is deactivated to its ground state. In the meantime, irradiation also leads to cofactor fragmentation, and FAP loses its activity, which is the inactivation process. We observed that the inactivated FAP results not only in the vanishing of the FAD fluorescence but also in the precipitating of the FAP enzymes. In previous studies under 455 nm light-emitting diode (LED) irradiation, photoinactivation processes related to a radical mechanism were proposed.^11,12^ These radical processes cause protein cross-linking and backbone cleavage. Interestingly, the *cv*FAP is more stable while forming an enzyme–substrate complex.^12^ These results indicate that the photoinactivation and photocatalytic processes compete with each other. In the presence of a substrate, the photocatalytic processes are dominant, and the photoinactivation processes are suppressed. Such photoinactivation processes were also observed in biocomposites (Figure 3B,C) with a slight decrease in phototoxicity. Although the ZIF could provide protective confinements to stabilize the enzyme structures and maintain the enzyme activity in harsh conditions, such as high temperature or high urea concentrations, when structural destruction originates from molecular fragmentation casing by the photoinduced radical reactions, such protective effects are limited.

**Figure 3:**
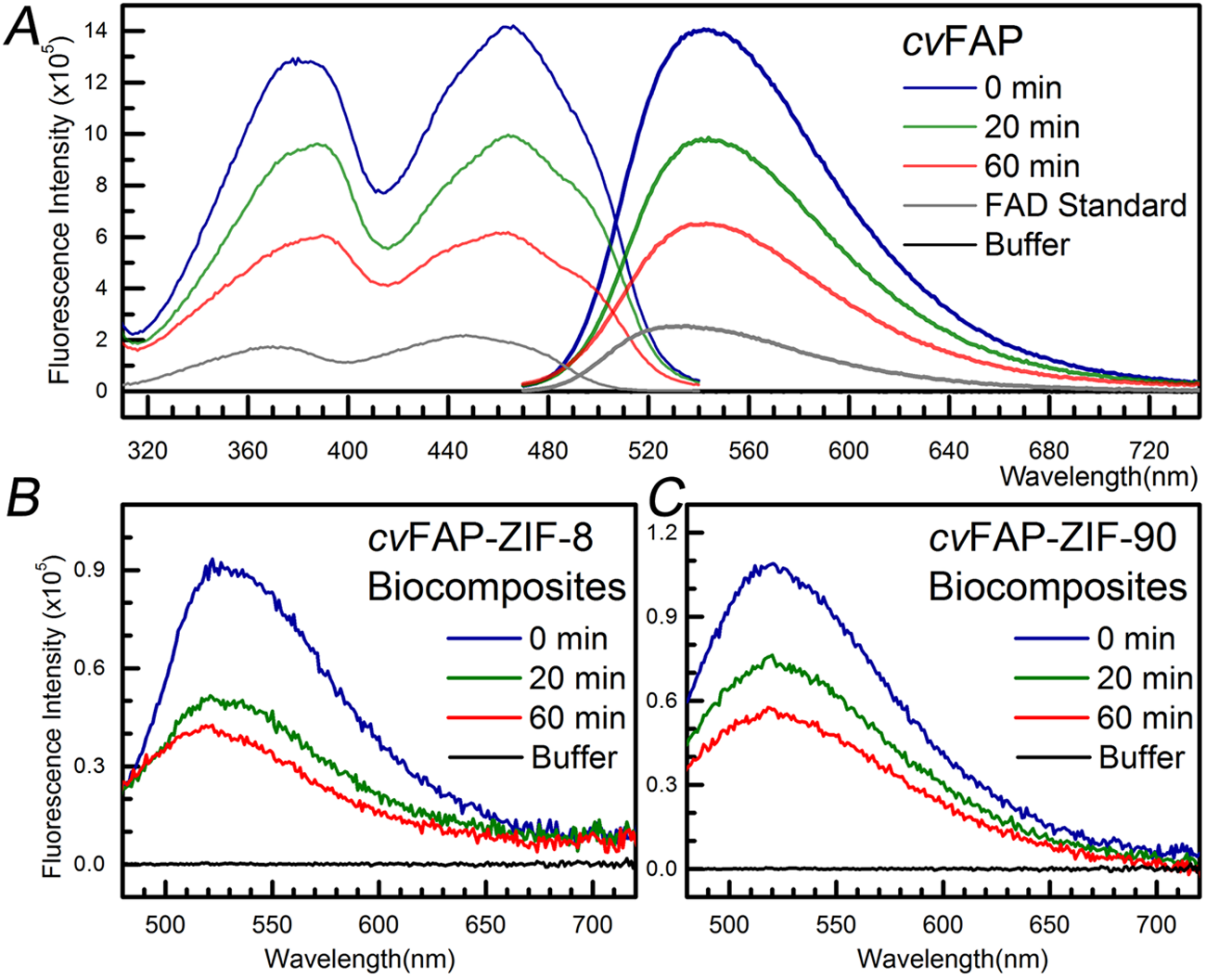
Blue-light-dependent inactivation of *cv*FAP in aqueous solution and two biocomposites. (A) The emission (bold line, 450 nm excitation) and excitation (thin line, 560 nm detection) spectra of *cv*FAP in aqueous solution after 450 nm irradiation for 0, 20, and 60 min. The gray trace represents the standard oxidized flavin FAD. (B, C) The emission spectra of *cv*FAP-ZIF-8 and *cv*FAP-ZIF-90 after 450 nm irradiation for 0, 20, and 60 min.

### 3.4 Photoinactivation and Deactivation

We carried out time-correlated single-photon counting measurements with *cv*FAP in aqueous solution and two biocomposites to further characterize the excited-state properties. Upon 450 nm excitation, the 560 nm emission was measured and analyzed. After purification, *cv*FAP in our studies did not include notable fatty acid substrates. Without a substrate, we observed a single-exponential decay of 4.2 ns (Figure 4A). It is the excited-state lifetime of the FAD cofactor without binding substrates and is close to previous observations measured after the consumption of all of the substrates.^2,9,10^ An additional and faster decay of 900–1000 ps was observed in the *cv*FAP-ZIF-8 and *cv*FAP-ZIF-90 biocomposites (Figure 4B,C), and their excited state exhibits faster dynamics with multiphase exponential decay of average lifetimes of 2.6 and 2.9 ns. In previous time-resolved serial femtosecond crystallography (SFX) studies, the FAP structure revealed a bent FAD conformation. The isoalloxazine ring of the FAD forms a bent butterfly conformation^29,30^ with the dihedral angle C4–N5–N10–C9 deviating from planarity of 11.0–17.4° in several measuring conditions.^9,34^ When *cv*FAP is at a low temperature of 100 K, the FAD is in the most bent conformation.^9^ Our steady-state spectra of the biocomposites indicate that the active site is more hydrophobic and possibly has fewer water molecules. Hence, we suppose that *cv*FAP enzymes are packed inside the ZIF cages, and the structure of the isoalloxazine ring of FAD could be altered to a more bent form. The bent form of the isoalloxazine ring leads to faster energy relaxation,^29,30^ resulting in the fast dynamics observed here, a lower yield in the inactivation route, and a more stable flavin cofactor.

**Figure 4:**
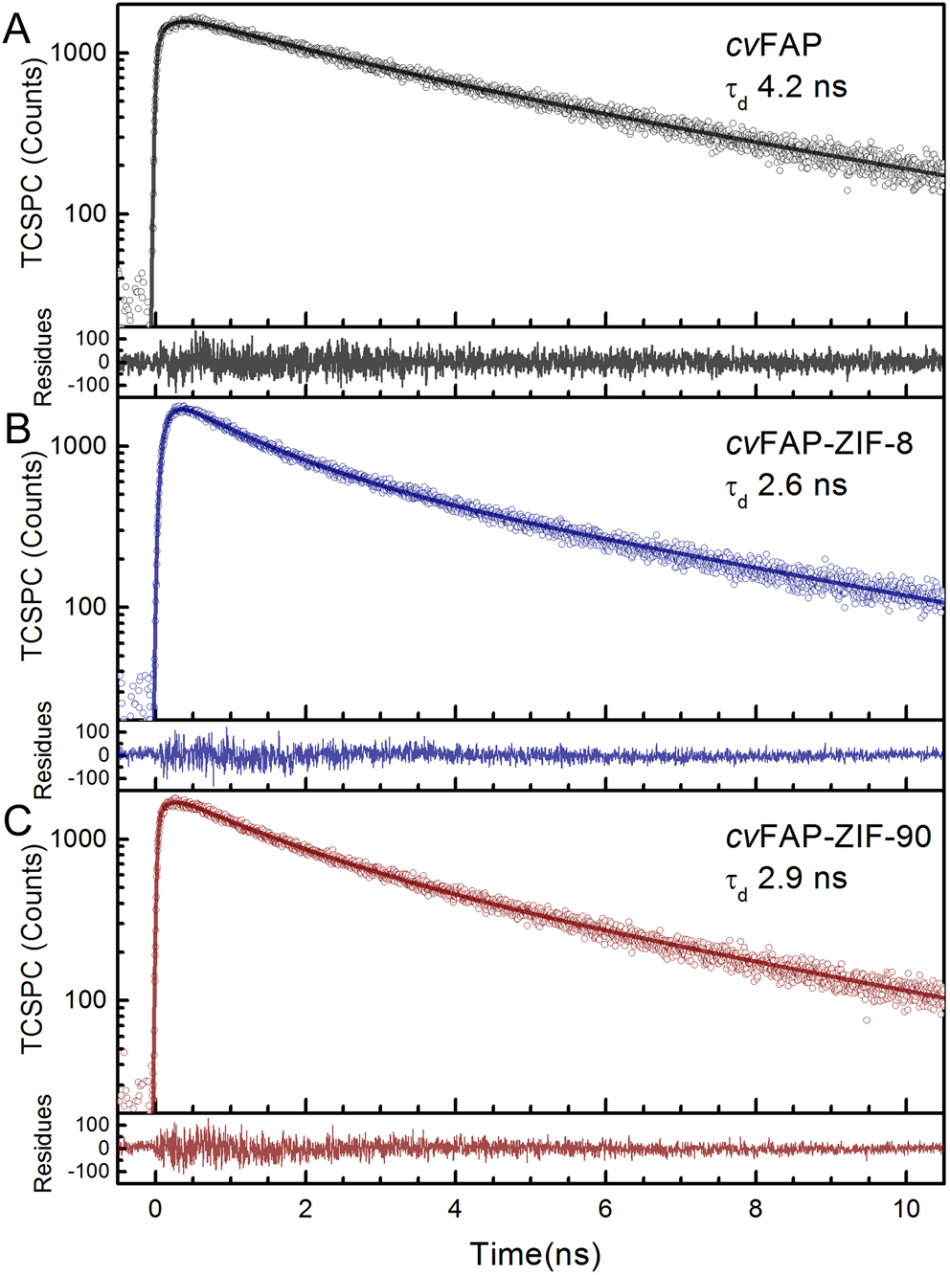
Time-resolved fluorescence decay measurements of *cv*FAP in aqueous solution (A) and two biocomposites (B, C) by TCSPC. Upon 450 nm excitation, the 560 nm emission was measured and analyzed as a sum of exponentials.

In *cv*FAP-ZIF-8 biocomposites, we observed similar dynamics in the absence and presence of the PA substrates, as shown in Figure S10A. These results indicate that no additional excited-state decay channel occurs in the presence of the PA substrates and further confirm the loss of activity resulting from the lack of enzyme–substrate complex formation in *cv*FAP-ZIF-8 biocomposites. However, in *cv*FAP in the reaction buffer and the *cv*FAP-ZIF-90 biocomposites, we observed additional faster decay dynamics of 0.42 ns (∼30% in total counts) and 0.55 ns (∼10% in total counts), respectively, as shown in Figure S10B,C. This additional faster decay indicates that the initial electron transfer from the substrate to the excited FAD is occurring.

## 4 Conclusions

In summary, we employed two different ZIF cages, ZIF-8 and ZIF-90, with *cv*FAP and characterized photocatalysis, photoinactivation, and excited-state dynamics of *cv*FAP in aqueous solution and biocomposites. Due to the preparation conditions and the surface properties, ZIF-8 forms a hydrophobic surface and shows a cluster-like packed morphology, and ZIF-90 forms a hydrophilic surface and shows a hexagon-like morphology. In both biocomposites, *cv*FAP exhibits a more dense and hydrophobic active site. Even though the conversion percentage is lower in *cv*FAP-ZIF-90 compared to *cv*FAP in the reaction buffers, the ZIF-90 cage protects the *cv*FAP from denaturing in higher temperature conditions. Thus, the environment’s disturbance in enzyme stability could be suppressed by the cage protection of ZIF-90.

Upon irradiation, the excited cofactor FAD undergoes energy deactivation to the ground state. Photon excitation also leads to FAD fragmentation and catalytic inactivation. An additional electron transfer channel occurs upon binding the substrate, and the photoproduct forms (Figure 5). The inactivation would be lessened in the presence of the substrates, in which the photocatalytic processes overwhelm the consequential protein fragmentation. In biocomposites, the deactivation processes are enhanced, resulting from a more bent cofactor structure and fast deactivation processes. However, although the ZIFs provide protective confinements to stabilize the enzyme structures, such protective effects are limited when structural destruction originates from molecular fragmentation casing by photoinduced radical reactions.

**Figure 5:**
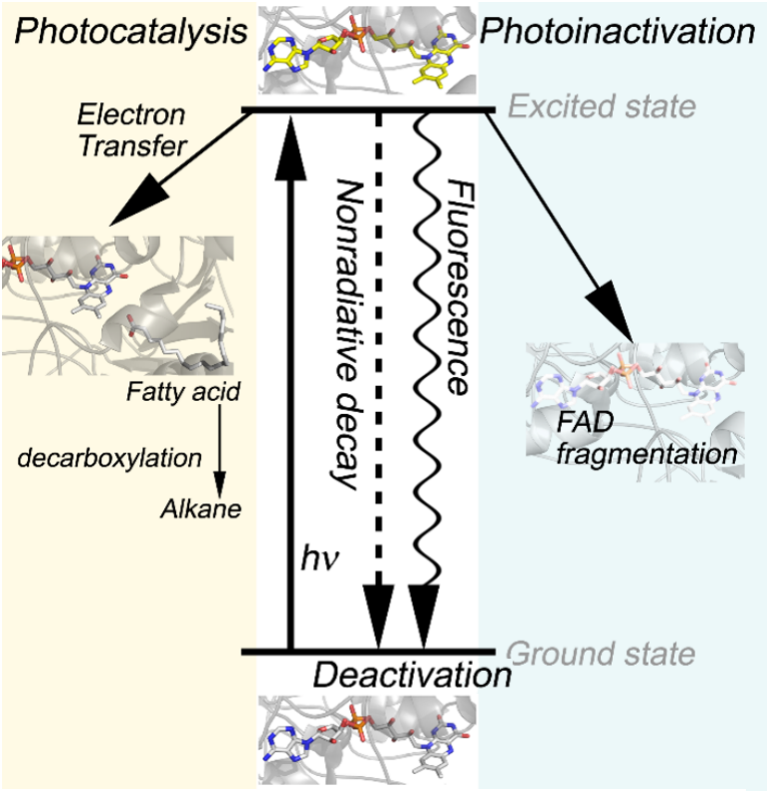
Schematic representation of photocatalysis, photoinactivation, and deactivation processes in *cv*FAP. Upon irradiation (hν), the FAD cofactor is excited from the ground state to the excited state. The excited ^1^FAD* cofactor undergoes energy relaxation and deactivates to its ground state (middle, white). Photon excitation also leads to FAD fragmentation, and FAP loses its catalytic function (right, light blue). An additional electron transfer channel occurs upon binding the substrate, and the photoproduct forms (left, light yellow). The *cv*FAP enzyme is represented in cartoon type, and the FAD cofactor and substrate are represented in stick type (PDB: 6ZH7).

## Supplementary Material





## Notes

Supplementary methods; schematic illustration of biocomposite synthesis (Figure S1); schematic illustration of the ACS-ACOD method (Figure S2); the steady-state spectra of *cv*FAP in aqueous solution and ZIF-90 cages (Figure S3); the morphologies of biocomposites by optical microscopy (Figure S4); the DIC and fluorescence images of the ZIF-90 cage from the multiphoton confocal microscopy sectioning image of the ZIF-90 cage (Figure S5); a classic SIM sectioning image of the *cv*FAP-ZIF-90 biocomposites from super-resolution microscopy (Zeiss Elyra 7) (Figure S6); the gas chromatography–mass spectrometry of standard palmitic acid, standard pentadecane, and *cv*FAP-PA complex under 450 nm irradiation of 0 and 4 h (Figure S7); the gas chromatography of 18.89 min GC retention time with EIC m/z256 for the Palmitic acid (Figure S8); the fluorescence emission spectra of *cv*FAP in aqueous solution and two biocomposites in the absence and presence of the PA substrates (Figure S9); the time-resolved fluorescence decay measurements of biocomposites at 560 nm, upon 450 nm excitation (Figure S10) (PDF)
Stacked classic SIM sectioning images (Movie S1) (GIF)

## References

[1] Sorigué D., Légeret B., Cuiné S., Morales P., Mirabella B., Guédeney G., Li-Beisson Y., Jetter R., Peltier G., Beisson F. (2016). Microalgae Synthesize Hydrocarbons from Long-Chain
Fatty Acids via a Light-Dependent Pathway. Plant
Physiol..

[2] Sorigué D., Légeret B., Cuiné S., Blangy S., Moulin S., Billon E., Richaud P., Brugière S., Couté Y., Nurizzo D., Müller P., Brettel K., Pignol D., Arnoux P., Li-Beisson Y., Peltier G., Beisson F. (2017). An Algal Photoenzyme Converts Fatty
Acids to Hydrocarbons. Science.

[3] Huijbers M. M. E., Zhang W., Tonin F., Hollmann F. (2018). Light-Driven Enzymatic
Decarboxylation of Fatty Acids. Angew. Chem.,
Int. Ed..

[4] Zhong D. (2007). Ultrafast
Catalytic Processes in Enzymes. Curr. Opin.
Chem. Biol..

[5] Kao Y.-T., Saxena C., Wang L., Sancar A., Zhong D. (2007). Femtochemistry
in Enzyme Catalysis: DNA Photolyase. Cell Biochem.
Biophys..

[6] Heyes D. J., Hardman S. J. O., Hedison T. M., Hoeven R., Greetham G. M., Towrie M., Scrutton N. S. (2015). Excited-State Charge
Separation in
the Photochemical Mechanism of the Light-Driven Enzyme Protochlorophyllide
Oxidoreductase. Angew. Chem., Int. Ed..

[7] Zhang S., Heyes D. J., Feng L., Sun W., Johannissen L. O., Liu H., Levy C. W., Li X., Yang J., Yu X., Lin M., Hardman S. J. O., Hoeven R., Sakuma M., Hay S., Leys D., Rao Z., Zhou A., Cheng Q., Scrutton N. S. (2019). Structural Basis for Enzymatic Photocatalysis in Chlorophyll
Biosynthesis. Nature.

[8] Heyes D. J., Lakavath B., Hardman S. J. O., Sakuma M., Hedison T. M., Scrutton N. S. (2020). Photochemical Mechanism
of Light-Driven Fatty Acid
Photodecarboxylase. ACS Catal..

[9] Sorigué D., Hadjidemetriou K., Blangy S., Gotthard G., Bonvalet A., Coquelle N., Samire P., Aleksandrov A., Antonucci L., Benachir A., Boutet S., Byrdin M., Cammarata M., Carbajo S., Cuiné S., Doak R. B., Foucar L., Gorel A., Grünbein M., Hartmann E., Hienerwadel R., Hilpert M., Kloos M., Lane T. J., Légeret B., Legrand P., Li-Beisson Y., Moulin S. L. Y., Nurizzo D., Peltier G., Schirò G., Shoeman R. L., Sliwa M., Solinas X., Zhuang B., Barends T. R. M., Colletier J.-P., Joffre M., Royant A., Berthomieu C., Weik M., Domratcheva T., Brettel K., Vos M. H., Schlichting I., Arnoux P., Müller P., Beisson F. (2021). Mechanism and Dynamics
of Fatty Acid Photodecarboxylase. Science.

[10] Wu R., Li X., Wang L., Zhong D. (2022). Ultrafast Dynamics and Catalytic
Mechanism of Fatty Acid Photodecarboxylase. Angew. Chem., Int. Ed..

[11] Lakavath B., Hedison T. M., Heyes D. J., Shanmugam M., Sakuma M., Hoeven R., Tilakaratna V., Scrutton N. S. (2020). Radical-Based Photoinactivation of Fatty Acid Photodecarboxylases. Anal. Biochem..

[12] Wu Y., Paul C. E., Hollmann F. (2021). Stabilisation of the Fatty Acid Decarboxylase
from *Chlorella variabilis* by Caprylic
Acid. ChemBioChem.

[13] Guo X., Xia A., Zhang W., Li F., Huang Y., Zhu X., Zhu X., Liao Q. (2023). Anaerobic
Environment as An Efficient Approach to Improve
the Photostability of Fatty Acid Photodecarboxylase. Chin. Chem. Lett..

[14] Liang K., Ricco R., Doherty C. M., Styles M. J., Bell S., Kirby N., Mudie S., Haylock D., Hill A. J., Doonan C. J., Falcaro P. (2015). Biomimetic
Mineralization of Metal-Organic
Framework as Protective Coatings for Biomacromolecules. Nat. Commun..

[15] Lyu F., Zhang Y., Zare R. N., Ge J., Liu Z. (2014). One-Pot Synthesis
of Protein-Embedded Metal-Organic Frameworks with Enhanced Biological
Activities. Nano Lett..

[16] Liao F.-S., Lo W.-S., Hsu Y.-S., Wu C.-C., Wang S.-C., Shieh F.-K., Morabito J. V., Chou L.-Y., Wu K. C.-W., Tsung C.-K. (2017). Shielding against
Unfolding by Embedding Enzymes in
Metal–Organic Frameworks via a *de Novo* Approach. J. Am. Chem. Soc..

[17] Shahsavari M., Mohammadzadeh Jahani P., Sheikhshoaie I., Tajik S., Aghaei Afshar A., Askari M. B., Salarizadeh P., Di Bartolomeo A., Beitollahi H. (2022). Green Synthesis of Zeolitic Imidazolate Frameworks:
A Review of Their Characterization and Industrial and Medical Applications. Materials.

[18] Pan Y., Liu Y., Zeng G., Zhao L., Lai Z. (2011). Rapid Synthesis of
Zeolitic Imidazolate Framework-8 (ZIF-8) Nanocrystals in An Aqueous
System. Chem. Commun..

[19] Shieh F.-K., Wang S.-C., Yen C.-I., Wu C.-C., Dutta S., Chou L.-Y., Morabito J. V., Hu P., Hsu M.-H., Wu K. C.-W., Tsung C.-K. (2015). Imparting Functionality to Biocatalysts
via Embedding Enzymes into Nanoporous Materials by a *de Novo* Approach: Size-Selective Sheltering of Catalase in Metal-Organic
Framework Microcrystals. J. Am. Chem. Soc..

[20] Tsai C.-K., Li J.-T., Ke S.-R., Li S.-H., Huang P.-Y., Chang C.-W. (2022). Preparation and
Application of Fluorescent Bioconjugates
in Probing the pH and Solvent Relaxation Dynamics in ZIF-8 Cavities. J. Phys. Chem. C.

[21] Tan B., Jiao Q., Zhang Y., Yan Y., Wang D., Li X., Zhu G., Fan J., Zhao H. (2024). Enzyme Encapsulation
into Zeolitic Imidazolate Framework-8/Graphene Oxide (ZIF-8/GO) for
Enhanced Stability and DNA-Mediated Catalytic Performance. Sens. Actuators, B.

[22] Shieh F.-K., Wane C.-S., Leo S.-Y., Wu C.-W. (2013). Water-Based
Synthesis
of Zeolitic Imidazole Framework-90 (ZIF-90) with a Controllable Particle
Size. Chem.—Eur. J..

[23] Kida K., Okita M., Fujita K., Tanaka S., Miyake Y. (2013). Formation
high crystalline ZIF-8 in an aqueous solution. CrysEngComm.

[24] Weng Y., Chen R., Hui Y., Chen D., Zhao C.-X. (2024). Boosting
Enzyme Activity in Enzyme Metal-Organic Framework Composites. Chem Bio Eng..

[25] Moulin S., Légeret B., Blangy S., Sorigué D., Burlacot A., Auroy P., Li-Beisson Y., Peltier G., Beisson F. (2019). Continuous Photoproduction of Hydrocarbon
Drop-In Fuel by Microbial Cell Factories. Sci.
Rep..

[26] Santner P., Szabó L. K., Chanquia S. N., Merrild A. H., Hollmann F., Kara S., Eser B. E. (2021). Optimization and
Engineering of Fatty
Acid Photodecarboxylase for Substrate Specificity. ChemCatChem.

[27] Öztürk N., Kao Y.-T., Selby C. P., Kavakli I. H., Partch C. L., Zhong D., Sancar A. (2008). Purification and Characterization
of a Type III Photolyase from *Caulobacter crescentus*. Biochemistry.

[28] Weber G. (1950). Fluorescence
of Riboflavin and Flavin-Adenine Dinucleotide. Biochem. J..

[29] Kao Y.-T., Tan C., Song S.-H., Öztürk N., Li J., Wang L., Sancar A., Zhong D. (2008). Ultrafast Dynamics
and Anionic Active States of Flavin Cofactor in Cryptochrome and Photolyase. J. Am. Chem. Soc..

[30] Kao Y.-T., Saxena C., He T.-F., Guo L., Wang L., Sancar A., Zhong D. (2008). Ultrafast Dynamics of Flavins in
Five Redox States. J. Am. Chem. Soc..

[31] Malicka J., Groth M., Karolczak J., Czaplewski C., Liwo A., Wiczk W. (2001). Influence of Solvents
and Leucine
Configuration at Position 5 on Tryptophan Fluorescence in Cyclic Enkephalin
Analogues. Biopolymers.

[32] Reichardt, C. Solvents and Solvent Effects in Organic Chemistry, 3rd ed.; Wiley-VCH: Weinheim, 2003.

[33] Stanley R.
J., MacFarlane A. W. (2000). Ultrafast Excited State Dynamics
of Oxidized Flavins: Direct Observations of Quenching by Purines. J. Phys. Chem. A.

[34] Hadjidemetriou K., Coquelle N., Barends T. R. M., De Zitter E., Schlichting I., Colletier J. P., Weik M. (2022). Time-Resolved Serial
Femtosecond Crystallography on Fatty-Acid Photodecarboxylase: Lessons
Learned. Acta Crystallogr., Sect. D: Struct.
Biol..

