# Supplementary material for: Characterization of Fatty Acid Photodecarboxylase in Zeolitic Imidazolate Frameworks
Source: ACS Omega. 2025 Aug 6;10(32):35595–603. doi: 10.1021/acsomega.5c01397 (PMC12368644; doi:10.1021/acsomega.5c01397)
Supplement: Supplementary file 1 [file ao5c01397_si_001.pdf]

Supplementary information

for

Characterization on Fatty Acid Photodecarboxylase in Zeolitic Imidazolate Frameworks

Min-Shih Su[1], *Ya-Ting Kao*[*1,2,3]

[1]Department of Biological Science and Technology, College of Engineering Bioscience, National Yang Ming Chiao Tung University, Hsinchu 30068, Taiwan.

[2]Institute of Bioinformatics and Systems Biology, College of Engineering Bioscience, National Yang Ming Chiao Tung University, Hsinchu 30068, Taiwan.

[3]Center For Intelligent Drug Systems and Smart Bio-devices (IDS^{2}B), National Yang Ming Chiao Tung University, Hsinchu 30068, Taiwan.

Supplementary Method and Results
Supplementary Figures: 1-10
Supplementary Movies: 1

Corresponding Author
*** Ya-Ting Kao,** E-mail: yatingkao@nycu.edu.tw

Supplementary Method

Classic SIM sectioning images

A schematic illustration of biocomposite synthesis is shown in Figure S1. The *cv*FAP-ZIF-90 biocomposite and ZIF-90 cage samples were resolved with the reaction buffer (50 mM Tris, 500 mM NaCl, and 50 % glycerol). ZIF-90 cage samples were loaded in a petri dish and were examined by optical microscopy with both differential interference (DIC) and confocal fluorescence images (Leica TCS-SP5-X AOBS). ZIF-90 cage samples were excited by a white light laser in the 470-480 nm range. The fluorescence images were measured by collecting 500-800 nm fluorescence through a 63X magnification objective. All images were enhanced with three superimposed scans and were processed by Leica LAS-AF-lite software. The image size is 82.54 μm × 82.54 μm with 6.79 μm Z-sectioning, and the pixel is 1280 × 1280. A section of both the fluorescent image and the bright field image of the ZIF-90 cages was taken. A droplet of The *cv*FAP-ZIF-90 biocomposite sample was sandwiched between two high-precision cover glasses with a thickness no. 1.5. Images were taken with the super-resolution microscope (Zeiss Elyra 7 with Lattice SIM^2) equipped with a 63X objective lens. Samples were excited at 488 nm, and fluorescent images were taken in the stacked mode with sCMOS camera of 55.9-ms camera exposure time. The acquired images were processed with classic SIM for enhanced resolution by the ZEN 3.0 microscopy software. A sacked sectioning image of the *cv*FAP-ZIF-90 biocomposites was taken.

Enzyme activity assay by the fluorometric method

The coupled enzymatic reaction system (the ACS-ACOD Method) was used to quantify non-esterified fatty acids (NEFA) in samples, as shown in Figure S2. First, acyl-CoA synthetase (ACS) catalyzes the fatty acids to acyl-CoA derivatives, fatty acyl-CoA. Then, in aerobic conditions, these derivatives are further converted to 2,3-trans-Enoly-CoA and hydrogen peroxide by acyl-CoA oxidase (ACOD). Finally, hydrogen peroxide reacts with the colorimetric probe by peroxidase (POD) for color development. The assay end-products could be measured by absorption and fluorescence emission spectroscopy. Under 535 nm excitation, the emission spectra were recorded. The fluorescence intensity directly reflects the amount of fatty acid remaining in the reaction mixture.

The gas chromatography and mass spectrometry

The *cv*FAP-substrate complex samples with the 450-nm irradiation time of 0 and 4 hours underwent heat shock processes for 10 min at 90 ˚C to stop the catalytic reaction and denature *cv*FAP enzymes. We performed multiple ether extractions to separate the remaining fatty acids and photoproducts from *cv*FAP and, more importantly, to remove DMSO. DMSO causes interference in GC-MS analysis and should be removed. Samples were further transferred to a centrifuge at 13000 rpm for 5 min, and the supernatants were collected. Finally, the solvents were further low-pressure dried.

Then, samples were re-dissolved in 100 μL ether and were heated by a multi-shot pyrolyzer (FRONTIER, EGA/PY-3030D) into the high-resolution gas chromatography (Agilent 7890B) and high-resolution mass spectrometry system (JEOL, AccuTOF GCX) with the separation column (Rxi-5MS) and Helium as carrier gas at a flow rate of 1 mL/min at 300°C after electron ionization.

Both standard palmitic acid (PA substrate, Sigma P5585) and standard pentadecane (PD photoproduct, Sigma P3406) also underwent gas chromatography and mass spectrometry (GC-MS) measurements to confirm their retention times in GC analysis.

<u>Supplementary Results</u>

The concentrations of compounds in solutions were determined by UV-VIS absorbance using the molar extinction coefficients: FAD_{OX} (ε_{467} =11300 $M^{-1}cm^{-1}$; ε_{280}=24300 $M^{-1}cm^{-1}$) and *cv*FAP (ε_{280} = 65695 $M^{-1}cm^{-1}$; ProtParam/ExPaSy; https://web.expasy.org/protparam/). A dilute *cv*FAP sample in the reaction buffer is shown in Figure S3A. Based on the absorbance at 280 nm and 467 nm, we estimate ~75% of the purified *cv*FAP contains the FAD cofactor. ZIF-8 cages are not fluorescent, but ZIF-90 cages exhibit a weak 520-nm peak emission, which partially overlaps with FAD emission. In Figure 2B, the fluorescence emission spectrum of *cv*FAP-ZIF-90 biocomposites is shown with the removal of the contribution from the ZIF-90 cage. A comparison of the spectra of *cv*FAP in the reaction buffer and ZIF-90 cages is shown in Figure S3B.

A section of both the fluorescent image and the bright field image of the ZIF-90 cages is shown in Figure S5. A sacked sectioning image of the *cv*FAP-ZIF-90 biocomposites was shown as a movie S1, and a single sectioning image at Z=23 is shown in Figure S6. Figures S5 and S6 indicate that the *cv*FAP enzymes are encapsulated in the ZIF-90 cages. Hence, cvFAP enzymes are embedded inside ZIF cages, not in the denatured forms.

The retention time of the PA substrate is 18.89 min, and that of the PD photoproduct is 13.69 min (Yellow labels in Figure S7A and Figure S7B), and the corresponding mass spectra and analyzed structures are shown in Figure S7A and Figure S7B. The results from gas

chromatography of the *cv*FAP-PA complex under 450-nm irradiation of 0 and 4 hours, and the corresponding mass spectra and analyzed structures are shown in Figure S6C and Figure S6D. From gas chromatography, these two peaks at 13.69 and 18.89 min displayed a mass spectrum identical to those of pentadecane and palmitic acid standards, respectively. Based on the gas chromatography of the *cv*FAP-PA complex under 450-nm irradiation for 0 and 4 hours, the decrease of PA and increase of PD were observed. The conversion percentages were estimated by the area under the corresponding PA-substrate retention as follows:

$$PA\ Conversion\ \% = \frac{(Area_{0-hr} - Area_{4-hrs})}{Area_{0-hr}} \times 100\%$$

The PA-to-PD conversions are estimated from the area under the GC peak at about 18.89 minutes (retention time) as shown in Figure S7. Due to the uncertainty of multiple extractions and drying, the GC-MS was used as a qualitative reference.

The *cv*FAP protein contains oxidized flavin (FAD_{OX}) as the cofactor. When excited by 450 nm irradiation, *cv*FAP is fluorescent and exhibits a fluorescence band with a peak of 550 nm and an FWHM of 100 nm. We prepared 1 μM *cv*FAP in a reaction buffer containing 40 mM Tris, 400 mM NaCl, 40 % glycerol, and 20 % DMSO at pH 8.0 with 0 and 200 mM PA substrate. Upon binding substrates, the FAD_{OX} fluorescence quenching was observed (Figure S9A). The fluorescence emission spectra of *cv*FAP in the reaction buffer and two biocomposites in the absence of PA substrates and the presence of the PA substrates (Figure S9B and S9C). With the presence of 200 μM PA substrate, we observed the fluorescence intensity decreases in *cv*FAP in the reaction buffer and the *cv*FAP-ZIF-90 biocomposite, but not in the *cv*FAP-ZIF-8 biocomposite.

- Movie S1: A stacked classic SIM sectioning image of the *cv*FAP-ZIF-90 from the super-resolution microscopy (Zeiss Elyra 7 with Lattice SIM[2]). Image size: 82.54 µm × 82.54 µm. Pixel: 1280 × 1280.

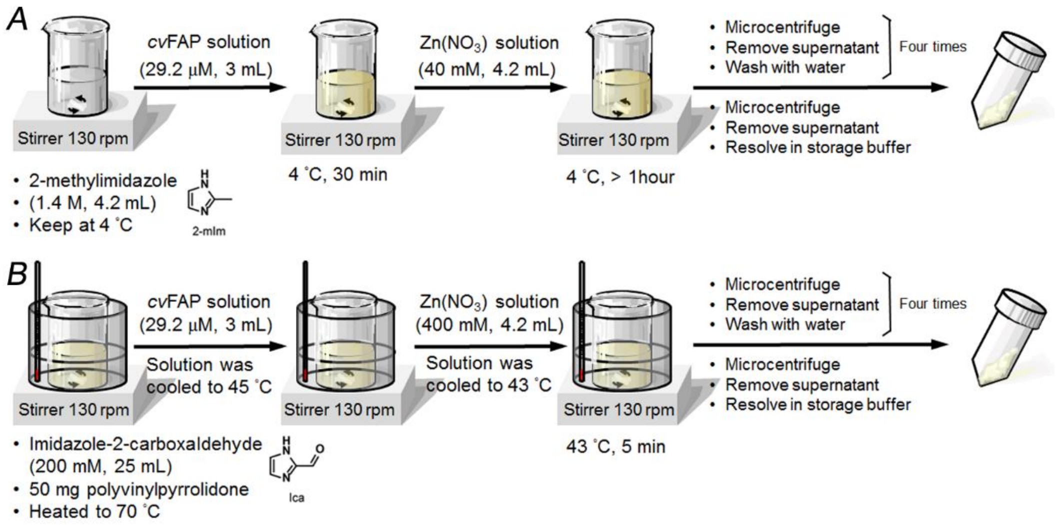

Figure S1: Schematic illustration of biocomposite synthesis. (A) *cv*FAP-ZIF-8. (B) *cv*FAP-ZIF-90.

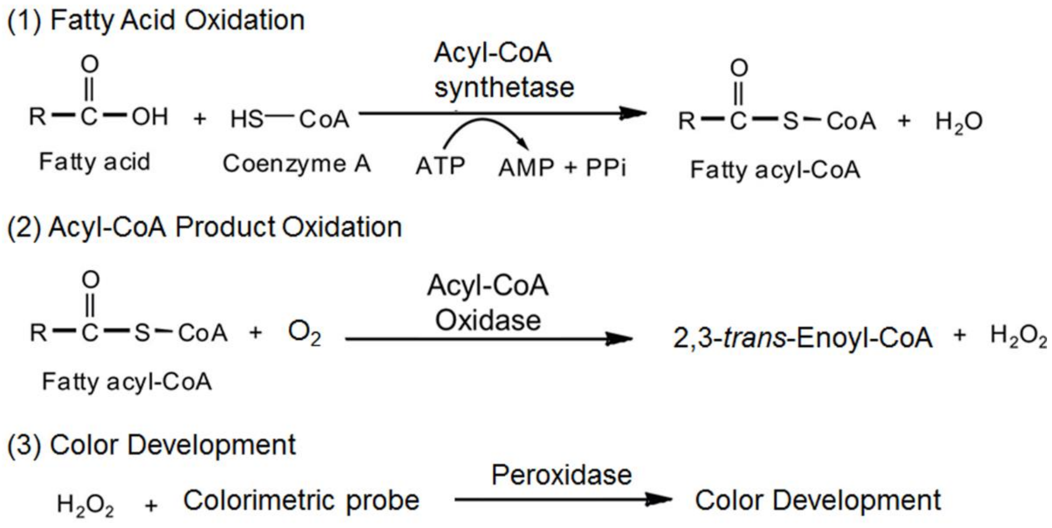

Figure S2: Schematic illustration of the ACS-ACOD method.

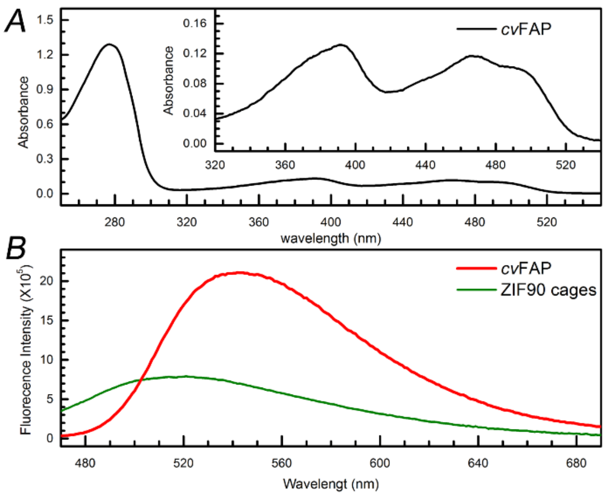

Figure S3: The steady-state spectra of *cv*FAP in aqueous solution and ZIF-90 cages. (A) A dilute *cv*FAP sample in the reaction buffer is shown. Based on the absorbance at 280 nm and 467 nm, we estimate ~75% of the purified *cv*FAP contains the FAD cofactor. (B) fluorescence emission spectra of *cv*FAP in the reaction buffer (red line) and pure ZIF-90 cages (green line). The emission peak of the ZIF-90 cage is 520 nm.

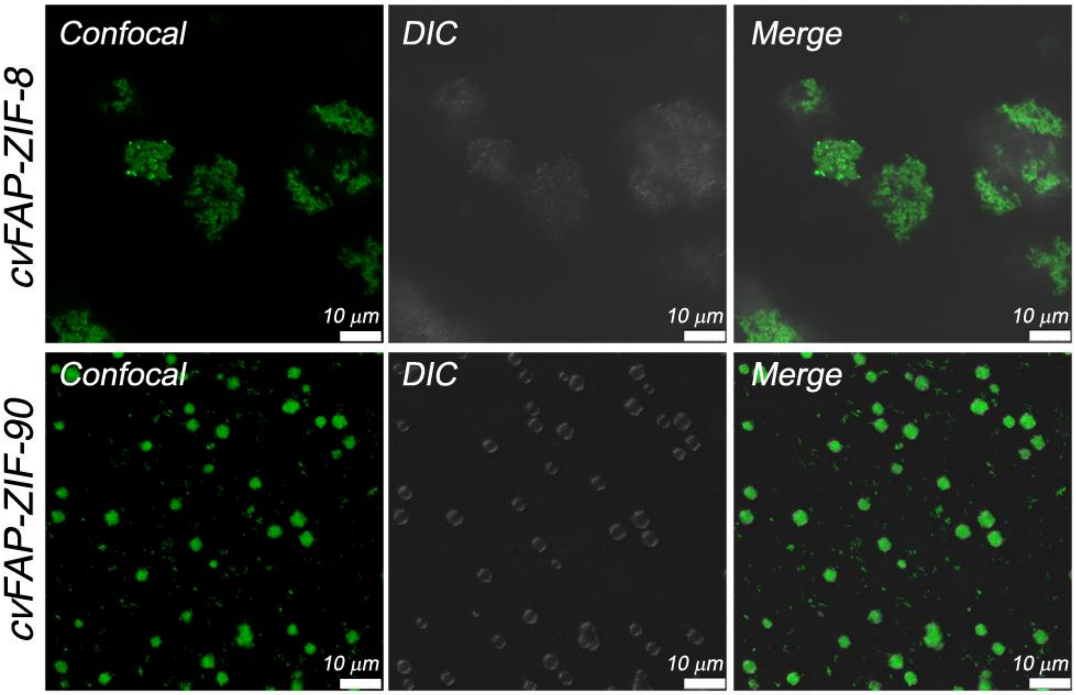

Figure S4: The morphologies of biocomposites by optical microscopy. The fluorescent (*Left*) images, DIC images(*middle*), and the merged (*Right*) images of the *cv*FAP-ZIF-8 (*upper*) and *cv*FAP-ZIF-90 (*Lower*) biocomposites.

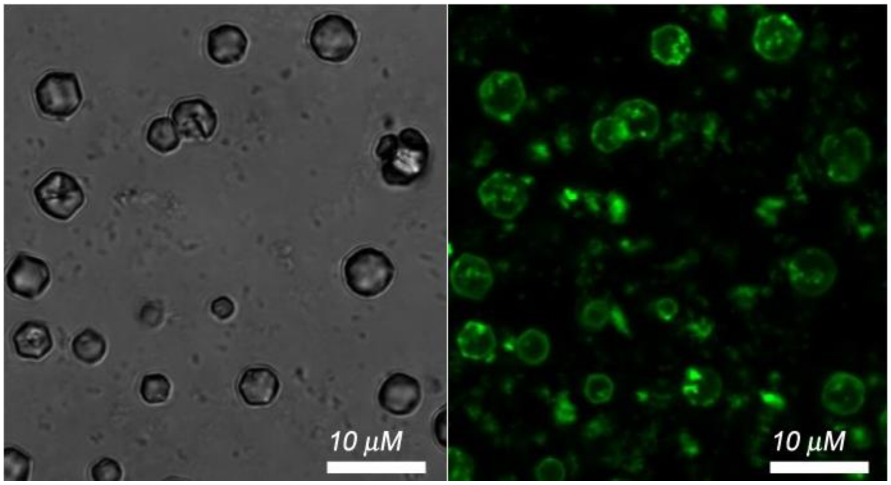

Figure S5: The DIC (*Left*) and fluorescent (*Right*) images of the ZIF-90 cage from the multiphoton confocal microscopy sectioning image of the ZIF-90 cage.

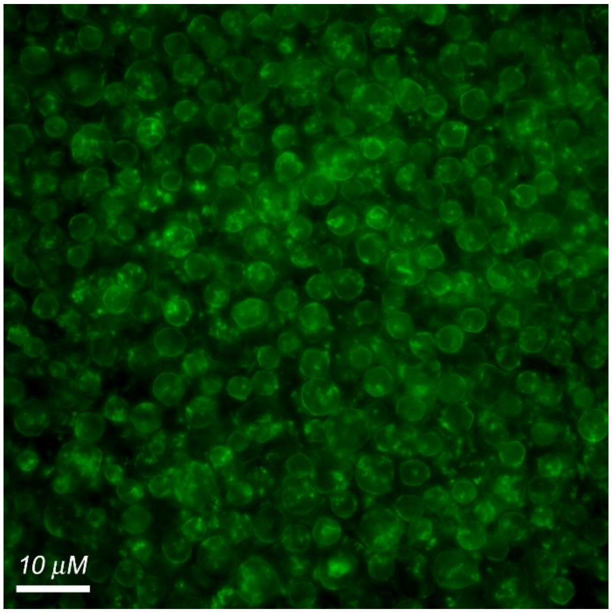

Figure S6: Classic SIM sectioning image of the *cv*FAP-ZIF-90 biocomposites from the super-resolution microscopy (Zeiss Elyra 7 with Lattice SIM[2]). Image size: 82.54 μm × 82.54 μm. Pixel: 1280 × 1280.

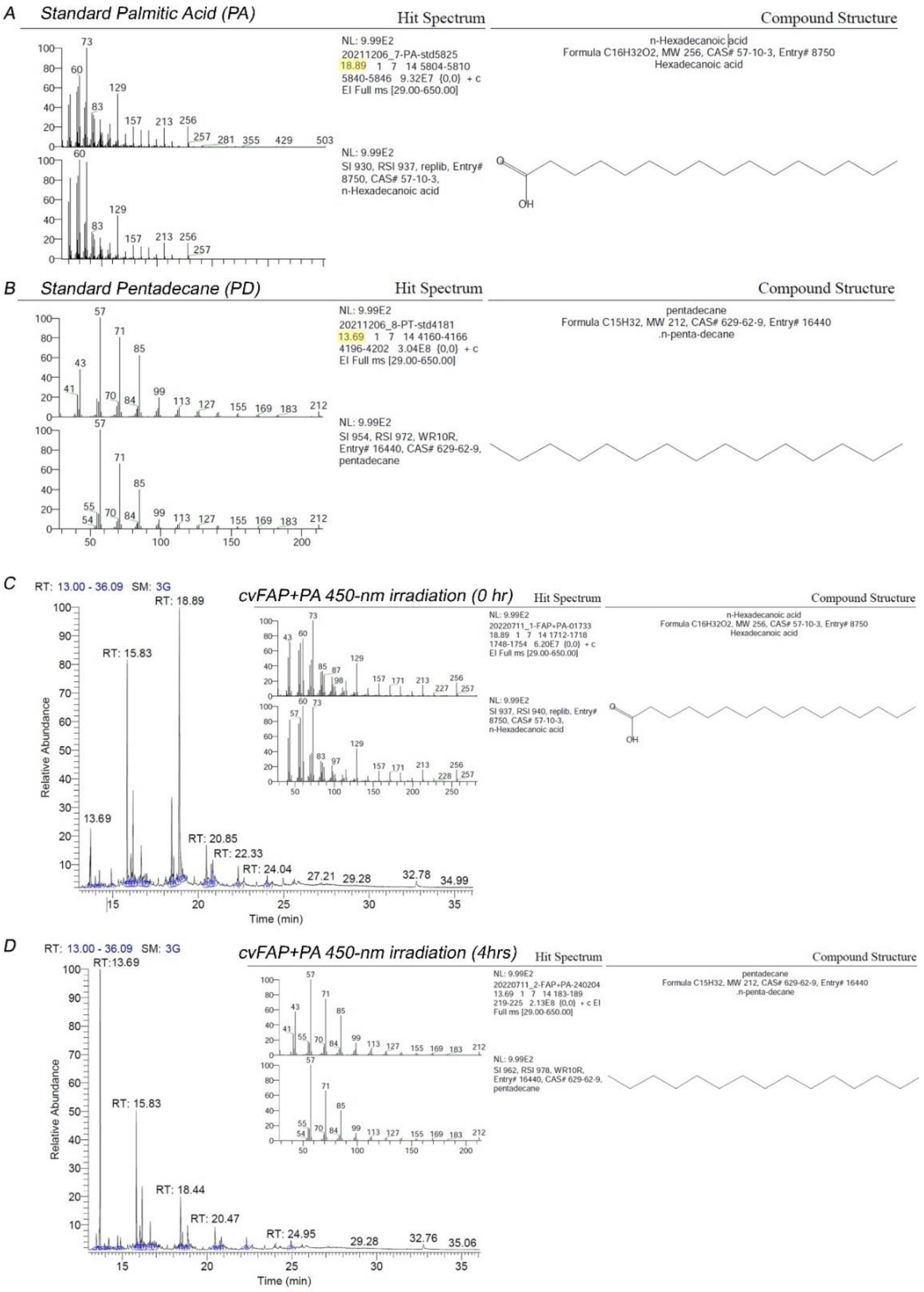

Figure S7: Gas chromatography and mass spectrometry. (A)The mass spectrometry of PA substrate at 18.89-min GC retention time. (B) The mass spectrometry of the PD photoproduct at 13.69-min GC retention time. (C) The gas chromatography of the *cv*FAP-PA complex before 450-nm irradiation. *Inset:* the mass spectrometry at 18.89 retention time. (D) The gas chromatography of the *cv*FAP-PA complex after 4 hrs 450-nm irradiation. *Inset:* the mass spectrometry at 13.69 retention time.

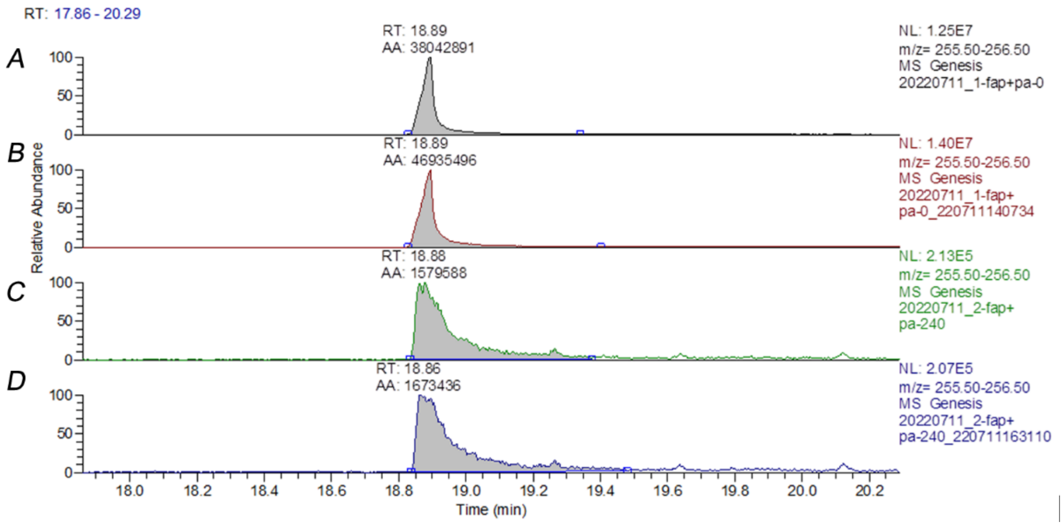

Figure S8: Gas chromatography of 18.89-min GC retention time with EIC m/z256 for the Palmitic acid. (A) and (B) *cv*FAP-PA complex in reaction buffer before 450-nm irradiation. (C) and (D) *cv*FAP-PA complex in reaction buffer after 4 hrs 450-nm irradiation. AA: area accumulation under corresponding retention time (RT)

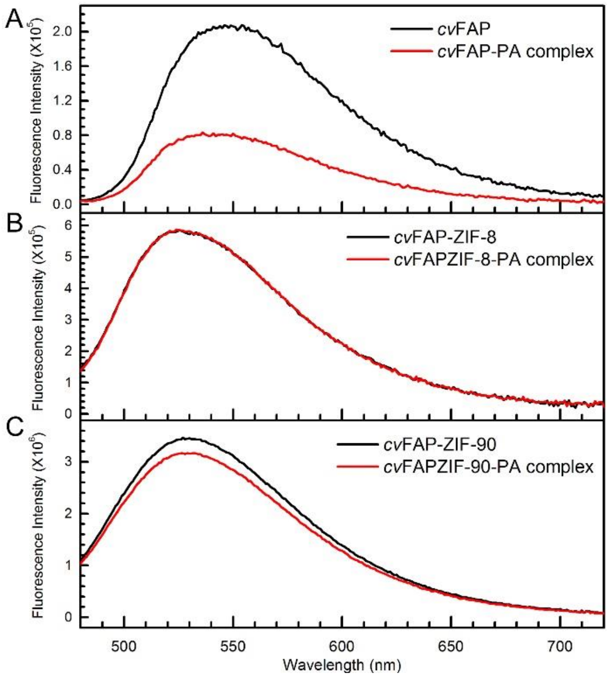

Figure S9: The fluorescence emission spectra of *cv*FAP in buffer and two biocomposites in the absence of PA substrates (black line) and the presence of the PA substrates (red line). (A) *cv*FAP in buffer. (B) *cv*FAP-ZIF-8 biocomposites. (C) *cv*FAP-ZIF-90 biocomposites.

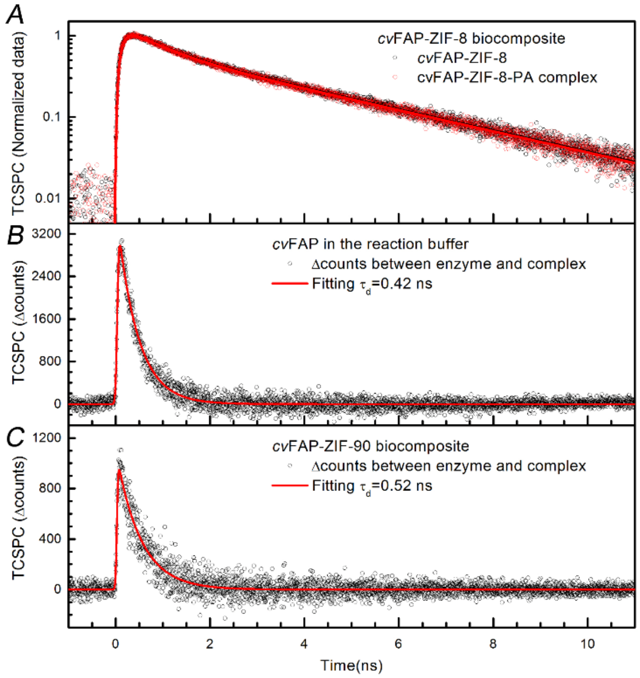

Figure S10: The time-resolved fluorescence decay measurements at 560 nm, upon 450 nm excitation. (A) *cv*FAP-ZIF-8 in the absence (black circles) and the presence (red circles) of the PA substrates. (B) The difference in TCSPC counts between the *cv*FAP-PA complex and the *cv*FAP enzyme in the reaction buffer. The faster decay of 0.42 ns was observed (~30%). (C) The difference in TCSPC counts between the *cv*FAP-ZIF-90-PA complex and the *cv*FAP-ZIF-90 biocomposite. The faster decay of 0.52 ns was observed (~10%).
