# Supplementary figures and images for: Characterization of Fatty Acid Photodecarboxylase in Zeolitic Imidazolate Frameworks
Source: ACS Omega. 2025 Aug 6;10(32):35595–603. doi: 10.1021/acsomega.5c01397 (PMC12368644; doi:10.1021/acsomega.5c01397)

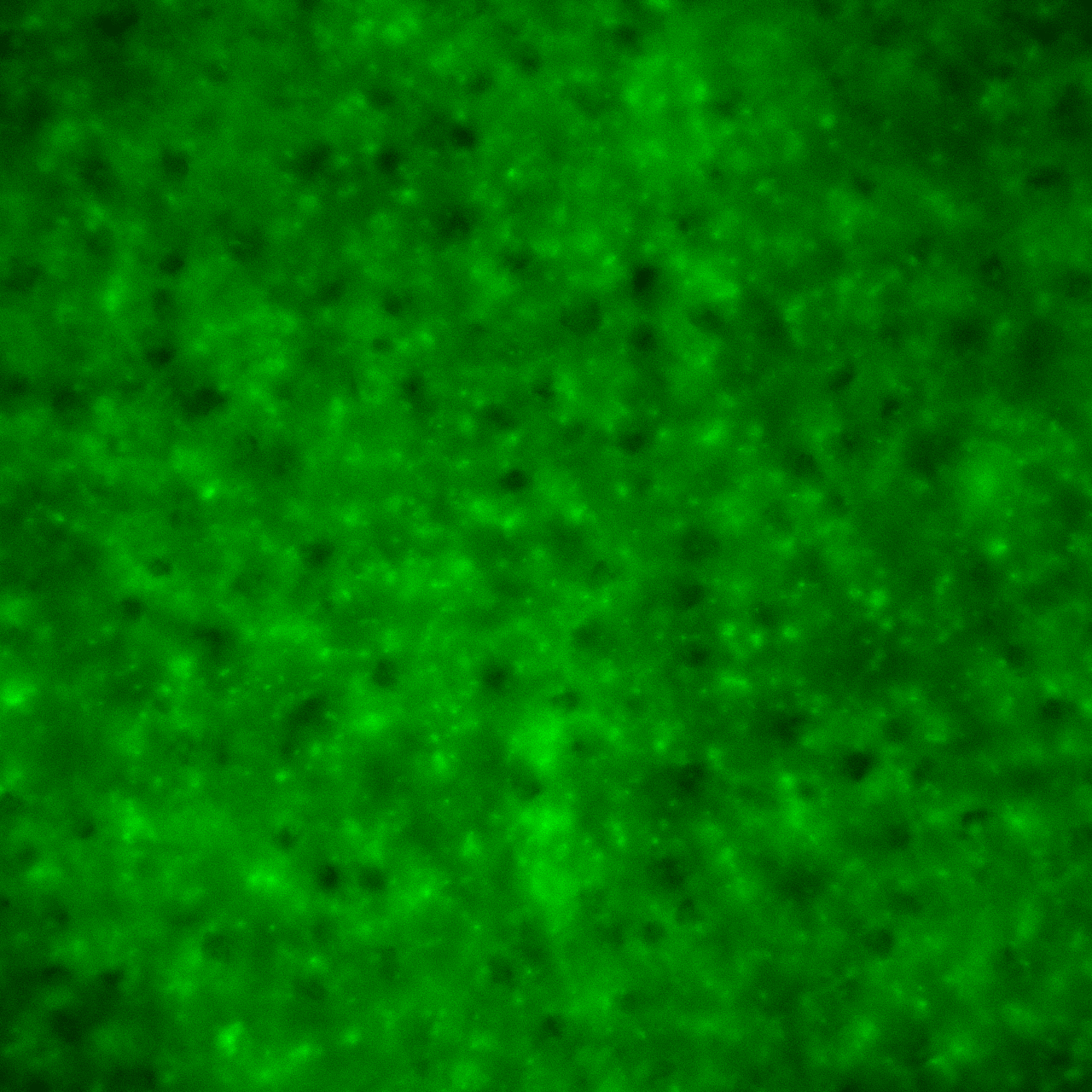

Supplement: Supplementary file 2 [file ao5c01397_si_002.gif]
